# Biosynthesis of Indole-3-Acetic Acid by New *Klebsiella oxytoca* Free and Immobilized Cells on Inorganic Matrices

**DOI:** 10.1100/2012/495970

**Published:** 2012-05-01

**Authors:** Valéria R. Celloto, Arildo J. B. Oliveira, José E. Gonçalves, Cecília S. F. Watanabe, Graciette Matioli, Regina A. C. Gonçalves

**Affiliations:** ^1^Departamento de Farmácia, Universidade Estadual de Maringá, Avenida Colombo 5790, 87020-900 Maringá, PR, Brazil; ^2^Centro Universitário de Maringá, Avenida Guedner 1610, 87050-390 Maringá, PR, Brazil

## Abstract

While many natural and synthetic compounds exhibit auxin-like activity in bioassays, indole-3-acetic acid (IAA) is recognized as the key auxin in most plants. IAA has been implicated in almost all aspects of plant growth and development and a large array of bacteria have been reported to enhance plant growth. Cells of *Klebsiella oxytoca* isolated from the rhizosphere of *Aspidosperma polyneuron* and immobilized by adsorption on different inorganic matrices were used for IAA production. The matrices were prepared by the sol-gel method and the silica-titanium was the most suitable matrix for effective immobilization. In operational stability assays, IAA production was maintained after four cycles of production, obtaining 42.80 ± 2.03 *μ*g mL^−1^ of IAA in the third cycle, which corresponds to a 54% increase in production in relation to the first cycle, whereas free cells began losing activity after the first cycle. After 90 days of storage at 4°C the immobilized cells showed the slight reduction of IAA production without significant loss of activity.

## 1. Introduction

Indole-3-acetic acid (IAA) is recognized as the key auxin in most plants and this phytohormone is critical for plant growth and orchestrates many developmental processes including cell enlargement and division, tissue differentiation, and responses to light and gravity [1, 2]. Diverse microorganisms including bacteria, fungi, and algae possess the ability to produce IAA. Interactions between IAA-producing bacteria and plants lead to diverse outcomes on the plant side, varying from pathogenesis to phytostimulation, and highlight the fact that bacteria use this phytohormone to interact with plants as part of their colonization strategy, including phytostimulation and circumvention of basal plant defense mechanisms [3].

Plant growth promoting rhizobacteria (PGPR) are a heterogeneous group of bacteria that can be found in the rhizosphere, at root surfaces and in association with roots, which can improve the extent or quality of plant growth directly and or indirectly. In last few decades a large array of bacteria including species of *Pseudomonas*, *Azospirillum*, *Azotobacter*, *Klebsiella*, *Enterobacter*, *Alcaligenes*, *Arthrobacter*, *Burkholderia*, *Bacillus*, and *Serratia* have been reported to enhance plant growth [4]. These bacteria from various crops synthesize and release IAA as secondary metabolites and this auxin produced by bacteria is proposed to act in conjunction with endogenous IAA in plant to stimulate cell proliferation, elongation, and enhancement of host's uptake of minerals and nutrients from the soil and act on microbial cell differentiation [3, 5, 6].

Immobilized biocatalysts such as enzymes, microorganisms, organelles, and plant and animal cells are being explored in the search for ways to increase yield in biotechnology processes [7]. Immobilizing microbial cells provides many benefits over free cells. The advantages of an immobilized system include their active metabolic ability to synthesize bioproducts and they can be handled more easily and recovered from the solution without difficulty [8]. Adsorption is the oldest and the easiest method for immobilization of biocatalysts in macroscopic water-insoluble carriers [9]. Sol-gel materials are growing in importance as solid supports for the immobilization. These materials have further advantages such as high homogeneity and purity, large surface area and porosity control, and well-modeled particles. Furthermore, inorganic materials, which are made mostly of metal oxides, are usually nontoxic, inert, and chemically and thermally stable [10–13].

The simplicity of the immobilization technique and the strength of the bond can help to develop future applications for the immobilization of whole cells [7]. The technology of immobilized cells of *Klebsiella oxytoca *has been studied to be applied in biological treatment to enhance the efficiency and effectiveness of biodegradation [14, 15]. Therefore, to the best of our knowledge, there are no reports on IAA production by immobilized cells of *K. oxytoca*.

Considering the economic importance of phytohormones, this study aimed to present the biosynthesis of IAA by a new strain of *K. oxytoca* and evaluate this biosynthesis by *K. oxytoca *free and immobilized cells on inorganic matrices by the sol-gel method.

## 2. Materials and Methods

### 2.1. Microorganism and Culture Conditions

The microorganism used in the current study was the bacterium *Klebsiella oxytoca*, which was isolated and identified in the Bacteriology Laboratory of the State University of Maringá, from the rhizosphere of *Aspidosperma polyneuron*. The bacteria cells were stored frozen (−6°C) in nutrient agar medium. For reactivation and production of cell biomass for immobilization, a medium composed of (g L^−1^) NaCl 10.0, tryptone 10.0, yeast extract 5.0 was used. The pH was adjusted to 7.0 with 0.1 N NaOH, and volumes of 100 mL were placed in 250 mL Erlenmeyer flasks that were autoclaved. Incubation was carried out with orbital shaking at 28°C for 48 h. Cells were harvested from the culture medium by centrifugation (5000 ×g, 20 min, 22°C) and the biomass was used for the necessary analyses.

### 2.2. IAA Production Medium

The culture medium used for IAA production was nitrogen-free malate (NFb) [16]. The medium was composed of K_2_HPO_4 _ (17.8 g L^−1^); KH_2_PO_4_ (159.5 g L^−1^); MgSO_4_·7H_2_O (0.2 g L^−1^); NaCl (0.1 g L^−1^); CaCl_2 _ (20 mg L^−1^); FeSO_4_·7H_2_O (20 mg L^−1^); Na_2_MoO_4_·2H_2_O (2.0 mg L^−1^); MnSO_4_·H_2_O (2.4 mg L^−1^); H_3_BO_3_ (2.8 mg L^−1^); CuSO_4_·5H_2_O (8.0 mg L^−1^); ZnSO_4_·7H_2_O (240 *μ*g L^−1^); biotin (100 *μ*g L^−1^). This medium was supplemented with 100.0 *μ*g mL^−1^ of *L*-tryptophan, and the pH was adjusted to 6.8 with 0.1 NaOH. The medium was divided into 100 mL Erlenmeyer flasks prior to sterilization.

### 2.3. Preparation of Matrices

To immobilize *K. oxytoca*, nine matrices were used: silica-titanium (6 and 11% SiO_2_/TiO_2_), silica-manganese (5 and 10% SiO_2_/MnO_2_), silica-titanium-antimony (6% SiO_2_/TiO_2_/Sb_2_O_5_), titanium-copper (TiO_2_/CuO), vanadium-titanium (V_2_O_5_/TiO_2_), and vanadium-silica (5 and 10% V_2_O_5_/SiO_2_).

The SiO_2_/TiO_2 _ matrix was prepared by adding 12.1 mL of an aqueous solution of 0.85 mol L^−1^ HNO_3_ in 250.0 mL of an ethanol solution of tetraethyl orthosilicate (TEOS) 50% (v/v). The mixture remained under reflux and shaking at 80°C for 150 min. Then, 21.0 mL of titanium (IV) tetrabutoxide (TBOT) and 490.0 mL of ethanol were added and the mixture was stirred for 2 h at room temperature. Sixty-six mL of an aqueous solution of 0.6 mol L^−1^ HNO_3 _ was slowly added and allowed to rest in order to gel. The xerogels formed were ground, dried at 110°C for 24 h, and sieved in order to obtain particles between 75 and 250 *μ*m diameter. The SiO_2_/TiO_2 _ binary oxide obtained was calcinated at 500°C under air flow [10, 17].

Based on the method used for synthesis of SiO_2_/TiO_2_, SiO_2_/MnO_2_ matrices were prepared, substituting TBOT for MnCl_2_. The V_2_O_5_/SiO_2 _ and V_2_O_5_/TiO_2_ matrices were prepared according to the method described by Colpini et al. [12], using HCl to hydrolyze TEOS, and HNO_3_ for TBOT, respectively. Mixed oxides were obtained through gelification with vanadium (V) oxide triisopropoxide (VOTIP).

To prepare the SiO_2_/TiO_2_/Sb_2_O_5_, antimony was adsorbed on the SiO_2_/TiO_2 _ matrix. To 15.0 g of the SiO_2_/TiO_2_ matrix were added 20.0 mL of 0.3 mol L^−1^ Sb (V) (prepared from SbCl_5_) solution (pH between 1 and 2) and 500.0 mL of distilled water. The suspension was heated at 60°C with agitation for 8 h. The solid was filtered, then washed with 1 mol L^−1^ HNO_3_ solution in order to avoid Sb (V) hydrolysis and to eliminate chloride ions, and finally washed in deionized water until the washing solution reached pH 4-5. The material was dried at 60°C for 96 h [10]. The TiO_2_/CuO matrix was prepared using TBOT and CuCl_2_.

The matrices obtained were characterized by X-ray fluorescence spectroscopy (XRFS), specific surface area (*S*_o_), average pore volume (*V*_p_), X-ray diffraction (XRD), and scanning electron microscopy (SEM) [10, 12].

### 2.4. Immobilization Procedure

 The method developed by Marsaioli et al. [18] for reduction of **β**-cetoester by immobilized *Serratia rubidaea* CCT 5732 cells on SiO_2_/TiO_2_ was modified and used in this study. An amount of 0.6 g of *K. oxytoca* whole cells with a known wet-cell weight (8.5 g dry cells/100 g wet cells) was used for each immobilization procedure. All immobilization processes were performed under aseptic conditions. Bacteria were immobilized in a 250 mL Erlenmeyer flask containing sterile distilled water (50.0 mL), *K. oxytoca* wet cells (0.6 g), and sterile support (0.3 g). The resulting suspension was shaken at 120 rpm and kept at 28°C for 24 h. After this time, the immobilized biomass was harvested from the medium washed with sterile distilled water and transferred to the IAA production medium.

### 2.5. Initial Biomass Effects

 The quantity of initial biomass for the process of immobilization and posterior IAA production was evaluated. Three quantities of initial biomass bacteria were tested, 0.3 g, 0.6 g, and 1.2 g of wet cells. For this assay, 6% SiO_2_/TiO_2 _ and 10% SiO_2_/MnO_2_ (0.3 g for each immobilization procedure) matrices were used. The culture medium for IAA production was supplemented with 1000.0 *μ*g mL^−1^ of *L*-tryptophan (the optimum amount to use without extraction). As it is in plants, *L*-tryptophan is also considered as an IAA precursor in bacteria because its addition to IAA-producing bacterial cultures increases IAA concentration in liquid medium [19, 20].

### 2.6. Evaluation of IAA Production by *Klebsiella oxytoca*

Cells immobilized in (6%) SiO_2_/TiO_2_ and free cells were centrifuged (5000 ×g, 20 min, 22°C) and submitted to an IAA production cycle. The assay was performed at 28°C, with shaking at 120 rpm for 48 h. The extraction technique used for IAA analysis was as follows: an aliquot of 30.0 mL of the supernatants were used, adjusted to pH 2.8, and 25.0 mL of ethyl acetate was added with magnetic agitation for 5 min. This procedure was carried out three times. Then, ethyl-acetate phases were rota-evaporated and then resuspended in 0.1 N NaOH. The production of IAA was estimated using Salkowski reagent [19–22].

### 2.7. Storage and Operational Stability

Four cycles of IAA production were carried out to determine operational stability. After each 48-h cycle, cells immobilized in 6% SiO_2_/TiO_2_ were removed from the production medium by centrifugation (5000 ×g, 22 min, 22°C), washed with sterile distilled water, and stored at 4°C until reutilization. For each cycle, a new IAA production medium was prepared and the IAA measured. Each cycle of IAA production was carried out for 48 h.

In order to evaluate storage stability, cells immobilized in 6% SiO_2_/TiO_2_ were divided into four aliquots and stored at 4°C. These aliquots were periodically sampled and submitted to IAA production cycles (zero time, 30, 60, and 90 days of storage) with no reutilizations.

Control cycles with free cells were also carried out in this assay. For comparison, the same aliquots (g) of free and immobilized cells were used. All experiments were carried out in triplicate.

### 2.8. Analytical Methods

#### 2.8.1. Scanning Electron Microscopy (SEM)

The condition of the microorganisms immobilized on mixed oxide support was evaluated by scanning electron microscopy. Immobilized cells were placed in a buffer solution (NaHPO_4_·7H_2_O/KH_2_PO_4_, 0.1 mol L^−1^ pH 7.0) with 2.5% glutaraldehyde for 24 h. After this period, the supernatant solution was discarded and the immobilized cells were washed three times with 30, 50, 70, 90, and 100% ethanol and left in absolute ethanol for further dehydration in a supercritical fluid extraction system using CO_2_ under high pressure. The same procedure was carried out with free cells. Samples of immobilized and free cells were deposited onto a double-face tape fixed to an aluminum sample holder and sputter-coated with a layer of gold in a BALZER MED 020. The photomicrographs were obtained with a scanning electron microscope (Shimadzu, model SS 550) with maximum accelerating voltage of 15 KV.

Using SEM photomicrographs (10000x), the number of bacterium cells per square meter (cells/m^2^) was calculated using the relative scale and the number of cells in the photomicrograph. The number of cells/g matrix was calculated by multiplying the number of cells/m^2^ by the respective matrix surface area.

#### 2.8.2. Spectrophotometric Quantification of Bacterial IAA

The IAA concentration was measured by the colorimetric method, using the Salkowski reagent. The assay was performed by mixing 1.0 mL of sample containing IAA with 2.0 mL of Salkowski reagent, containing 7.9 M H_2_SO_4_ with 1.2 g FeCl_3_, and after 25 min the absorbance was measured at 530 nm using a Varian Cary Spectrophotometer (Model 100 1 E UV-VIS). For the blank, the sample was replaced by 0.1 N NaOH. A standard curve was drawn for comparison, to determine auxin production by *K. oxytoca*.

### 2.9. Statistical Analysis

 The results were submitted to analysis of variance (ANOVA) and Tukey means tests, with a 5.0% level of probability, using the software Statistica 6.0/2001 (Stat Soft, Inc. Tulsa, OK, EUA).

## 3. Results and Discussion

### 3.1. Support Characterization

Table 1 shows the results of the chemical analyses of the specimens obtained as measured by X-ray fluorescence spectroscopy, the values of the specific surface areas (*S *_o_), and the average pore volume (*V*_p_). The quantities of titanium, antimony, manganese, vanadium, and copper in the SiO_2_/TiO_2_, SiO_2_/TiO_2_/Sb_2_O_5_, SiO_2_/MnO_2_, V_2_O_5_/TiO_2_, V_2_O_5_/SiO_2_, and TiO_2_/CuO matrices were proportional to the precursor reagent added in the experimental part.

 The decrease of the specific surface area is presumably related to the blocking of the finest pores upon Sb_2_O_5_ loading of the SiO_2_/TiO_2_ matrices [10]. Characteristics associated with the sol-gel method explain the results shown in Table 1. The exceptionally high specific surface area measurements of synthesized mixed oxide indicate a highly porous structure, which was confirmed by SEM photomicrographs [10, 12].

 The scanning electron photomicrographs of the matrices showed that Ti, Sb, Mn, V, and Cu were, under the magnification used, homogeneously distributed in the samples, with no detectable agglomeration of the metal-oxide particles separated phase.

X-ray diffraction analysis revealed a nearly amorphous phase, but with some diffraction lines [10, 12]. This indicates effective dispersion of Ti, Sb, Mn, V, and Cu in these mixed oxides. The analysis of the results for mixed oxides showed that these materials are porous and have a high surface area, and they also show high thermal stability and mechanical resistance, essential characteristics that make them good candidates for cell immobilization.

### 3.2. Microbial Cell Immobilization

**Figure 1:**
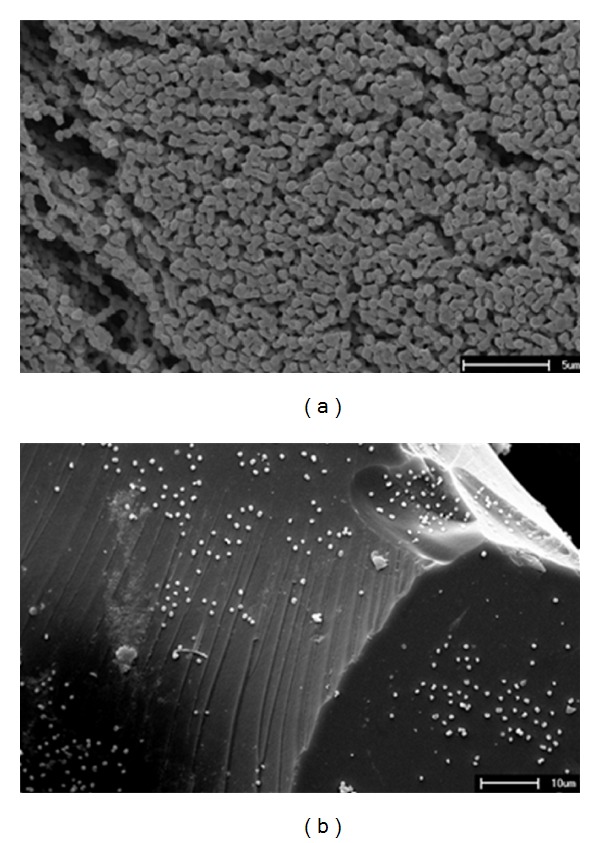
Scanning electron micrographs. (a) Free cells of* Klebsiella oxytoca* (3000x). (b) Immobilized cells on 6% SiO_2_/TiO_2_ (1000x).

Immobilization of *K. oxytoca* in the matrices was evaluated through scanning electron microscopy (SEM) photomicrographs.  Figure 1(a) shows SEM photomicrographs of free cells of *K. oxytoca*. The size of the bacterium was estimated as 0.4 *μ*m × 0.7 *μ*m.

 Considering that the mixed oxide had a smaller mean pore volume than the diameter of the bacterium, it can be assumed that there was no possibility that the microorganisms would penetrate into the matrices, meaning that the cells were immobilized by adsorption on their surface.

From the results in Table 2, (6%) SiO_2_/TiO_2_ matrix was better supports than the others that were tested, showing high average surface density of bacterial cells per square meter of support and high numbers of cells per gram of matrix. Based on this result, the (6%) SiO_2_/TiO_2_ matrix was chosen for the assessments of operational stability and storage stability.

After the bacteria were immobilized on the support, the SEM photomicrographs showed that they were adsorbed on the entire support and no alteration in cell morphology was observed after the adsorption (Figure 1(b)). The support was apparently not toxic to the cells, because no alteration in cell morphology was observed after the adsorption.

No differences in the immobilization were observed when different amounts of metal were utilized in the matrices (Table 2), for example, 6% and 11% SiO_2_/TiO_2_, 5% and 10% SiO_2_/MnO_2_, and 5% and 10% V_2_O_5_/SiO_2_. The reason for this lack of differences may be that increasing the concentration of metal reduced the surface area.

### 3.3. Initial Biomass Effect

**Figure 2:**
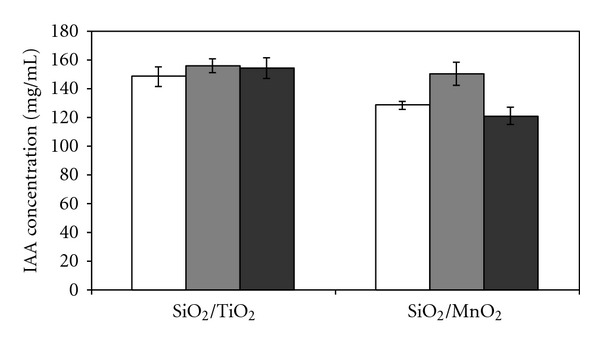
Initial biomass effect on IAA production by immobilized cells of* Klebsiella oxytoca* on 6% SiO_2_/TiO_2_ and 10% SiO_2_/MnO_2_ matrices ((white bar) 0.3 g, (grey bar) 0.6 g, and (black bar) 1.2 g of immobilized cells). Conditions: nitrogen-free malate (NFb) medium, 1000.0 *μ*g mL^−1^ of *L*-tryptophan, pH 6.8, 28°C, and 120 rpm.

The amount of biomass in the immobilization was an important parameter that influenced the production of IAA by immobilized cells. Data were analyzed by factorial analysis of variance (ANOVA) with the contrast test using the Statistica 8.0 program. All hypotheses were tested at the 95% confidence level. The results are presented as the mean ± standard deviation (Figure 2). In this assay, 1.0 mg mL^−1^ of *L*-tryptophan was added to the culture medium for IAA production. For the IAA analysis, the supernatants were analyzed directly, without extraction. The effects of different concentrations of *L*-tryptophan were studied by Shokri and Emtiazi [23] and revealed that maximum growth and IAA production were observed at 3.0 mg mL^−1^ *L*-tryptophan and more concentration (4.5 mg mL^−1^) causes reduction in IAA production. This data revealed that there is an optimum amount of *L*-tryptophan for maximum production of IAA and there is no linear relation between IAA production and amount of *L*-tryptophan.

For the (6%) SiO_2_/TiO_2_ matrix, the increase of the initial biomass from 0.3 to 0.6 g led to an increase of IAA production by 6%, reaching a production of 152.94 ± 1.91 *μ*g mL^−1^. IAA production remained statistically the same when 0.6 and 1.2 g were used, which may indicate that the space for further immobilization on the support had become saturated. For cells immobilized on (10%) SiO_2_/MnO_2_, IAA production was 18% higher when the amount of initial biomass was increased from 0.3 to 0.6 g, reaching 146.08 ± 2.58 *μ*g mL^−1^ (Figure 2). These results showed that the optimum amount of initial biomass for the immobilization procedure, for the highest IAA production, was 0.6 g of *K. oxytoca *for both matrices tested.

### 3.4. Operational Stability

The main advantage of immobilization is the ease of separation of cells from the production medium for possible reuse, together with the ease of storage and transportation. These attributes make the immobilized microorganism similar to a workbench chemical that can be transported (marketed) in a simple way and reused without the need for previous treatment [12, 24].

**Figure 3:**
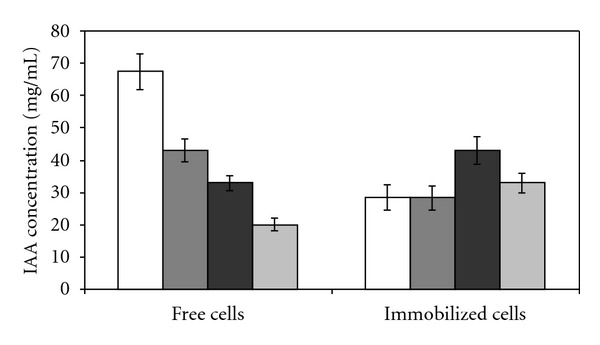
Operational stability of the free and immobilized cells of *Klebsiella oxytoca* on 6% SiO_2_/TiO_2_ (first cycle (white bar), second cycle (dark grey bar), third cycle (black bar), and fourth cycle (light grey bar)). Conditions: nitrogen-free malate (NFb), 100.0 *μ*g mL^−1^ of *L*-tryptophan, pH 6.8, 28°C and 120 rpm.

In this assay, 100.0 *μ*g mL^−1^ of *L*-tryptophan was added to the culture medium for IAA production, and for IAA analysis an extraction procedure was carried out. The extraction improved the efficiency of IAA detection. The reusability of *K. oxytoca *cells immobilized in (6%) SiO_2_/TiO_2_ was evaluated in four repeated cycles of 48 h each and was compared with free cells, which were reactivated over a period of 24 h (Figure 3). Each replacement of the production medium was designated as a reuse.

In the first cycle, IAA production was better for free cells (67.27 ± 1.06 *μ*g mL^−1^) than for the immobilized cells (27.79 ± 0.03 *μ*g mL^−1^). However, in the subsequent cycles, the production of the free cells decreased sharply, whereas for the immobilized cells the IAA production remained constant. The lower production of IAA by the immobilized cells in the first cycle probably occurred because of the lower aeration of the immobilized cells. Sedimentation of the cells occurred because of the weight of the matrix, even with agitation on an orbital shaker [25]. The amount of production with an immobilized system depends upon the nature of the material matrix, which affects the permeability of the cell to high penetration of the substrate and allows more rapid removal of the end products from the fermentation sites. The material matrix protects the bacterium from microenvironmental changes, thus producing unfavorable conditions for diffusion of media nutrients to the immobilized cells and limiting the transportation of oxygen to the inside of the biocatalyst, lowering the efficiency of the biocatalyst [7].

The free cells showed a decrease in production from the second cycle on, falling to 42.96 ± 1.68 *μ*g mL^−1^ of IAA. For the cells immobilized on (6%) SiO_2_/TiO_2_, the best result was achieved in the third cycle, obtaining 42.80 ± 2.03 *μ*g mL^−1^ of IAA, which corresponds to a 54% increase in production in relation to the first cycle. Similar results were observed by Mazzer et al. [26], where the maximum production of *β*-cyclodextrin by cells of *Bacillus firmus,* immobilized on calcium alginate, was obtained in the third cycle of production.

In the third cycle the production of IAA by immobilized cells was 28% higher than the amount of IAA produced by free cells. Cells of *K. oxytoca* immobilized on (6%) SiO_2_/TiO_2_ maintained almost the initial activity after four cycles of IAA production (Figure 3), which indicates that they can be repeatedly reused. This suggests that the immobilized cells formed a stable system for IAA production. Also, IAA productivity by the immobilized cells (163.30 U/mL at 8 days) was higher than that of free cells (132.40 U/mL at 8 days).

### 3.5. Storage Stability

The long-term viability and continued metabolic activity are among the most important advantages of working with an immobilized system. The extended viability of the immobilized cells may result from their different protein, nucleic acid, and inorganic substance compositions, compared to free cells [25].

Storage stability was evaluated for cells immobilized on the (6%) SiO_2_/TiO_2_ matrix and free cells kept at 4°C in a refrigerator for 90 days. The best operational stability was obtained with the immobilized cells. The second cycle showed approximately the same IAA production obtained in the first cycle (33.38 ± 0.90 *μ*g mL^−1^), and 94% and 83% production was maintained in the third and fourth cycles, respectively. The activity of the free cells decreased sharply after thirty days of storage and they were completely inactive in the third cycle. The results showed that the immobilized cells were quite stable, and after 90 days of storage they show slight reduction of IAA production in relation to newly immobilized cells. This result concords with the findings of Moriwaki et al. [9], where *Bacillus firmus* immobilized in inorganic matrices of SiO_2_/TiO_2 _ and SiO_2_/MnO_2_ showed the maximum production of *β*-cyclodextrin after 90 days of storage at 4°C.

## 4. Conclusions

Diverse bacterial species possess the ability to produce the IAA and in this study the biosynthesis of IAA by new *Klebsiella oxytoca* immobilized cells on SiO_2_/TiO_2_ matrix showed stability, with no significant loss of activity after 90 days of storage and with the possibility to expand the IAA production to four cycles, within the assay conditions studied. Considering that IAA has since been implicated in almost all aspects of plant growth and development, it is necessary to further optimize the fermentation conditions (use of bioreactors, culture medium modifications, temperature) to satisfy the requirements for large-scale IAA production from this potent isolate.

## Figures and Tables

**Table 1:** Chemical analyses (XRFS), specific surface area (*S*_o_), and pore volume (*V*_p_) of the mixed oxide.

Matrix	Ti (wt%)	Sb (wt%)	Mn (wt%)	V (wt%)	Cu (wt%)	*S*_o_ (m^2 ^g^−1^)	*V*_ p_ (mL g^−1^)
SiO_2_/TiO_2_ (6%)	6.2					705	0.44
SiO_2_/TiO_2_ (11%)	12.1					596	0.46
SiO_2_/TiO_2_/Sb_2_O_5_ (6%)	5.6	7.4				538	0.43
MnO/SiO_2_ (5%)			6.2			705	0.44
MnO/SiO_2_ (10%)			12.1			593	0.46
TiO_2_/CuO					3.25	37.8	0.08
TiO_2_/V_2_O_5_	5.9					436	0.10
V_2_O_5_/SiO_2 _ (5%)				5.66		468	0.20
V_2_O_5_/SiO_2 _ (10%)				11.74		379	0.16

**Table 2:** Surface densities and quantity of bacterial cells on the support.

Matrix	Bacterium cells/m^2^	Cells/g matrix
SiO_2_/TiO_2_ (6%)	4.79 × 10^11^	3.38 × 10^14^
SiO_2_/TiO_2_ (11%)	3.60 × 10^11^	2.14 × 10^14^
SiO_2_/TiO_2_/Sb_2_O_5 _ (6%)	4.19 × 10^11^	2.26 × 10^14^
MnO/SiO_2_ (5%)	3.60 × 10^11^	2.53 × 10^14^
MnO/SiO_2_ (10%)	3.99 × 10^11^	2.15 × 10^14^
TiO_2_/CuO	5.99 × 10^11^	2.26 × 10^13^
TiO_2_/V_2_O_5_	2.39 × 10^11^	9.56 × 10^12^
V_2_O_5_/SiO_2 _ (5%)	0.599 × 10^11^	2.80 × 10^13^
V_2_O_5_/SiO_2 _ (10%)	1.67 × 10^11^	6.33 × 10^13^
